# The Socioeconomic Impact of Surgical Site Infections

**DOI:** 10.3389/fpubh.2021.712461

**Published:** 2021-08-04

**Authors:** Emmanuel Piednoir, Joan Robert-Yap, Patrice Baillet, Emilie Lermite, Niki Christou

**Affiliations:** ^1^Centre de prévention des infections associées aux soins, University Hospital Rennes, Rennes, France; ^2^Department of Visceral Surgery, Geneva University Hospital and Medical School, Geneva, Switzerland; ^3^Visceral Surgery, Clinique du Parisis, Cormeilles-en-Parisis, France; ^4^Endocrine and visceral surgery department, University Hospital Angers, Angers, France; ^5^General Surgery Department, University Hospitals Birmingham NHS Foundation Trust, Birmingham, United Kingdom; ^6^Digestive Surgery Departement, University Hospital Limoges, Limoges, France

**Keywords:** surgical site infection, economic impact, burden, digestive surgery, worldwide

## Abstract

**Précis:** Surgical site infections are an ever-increasing phenomenon worldwide due to different factors. This brief report aimeds to highlight at a glance, for both physicians and political and institutional leaders, the economic burden of surgical site infections.

**Objectives:** This brief report aimed to highlight the economic burden of surgical site infections (SSIs).

**Methods:** A narrative review focusing on this subject has been carried out.

**Results:** Surgical site infections are responsible for generating important costs. In 2017, a French cohort highlighted a mean cost of each SSI treatment to be around €1,814; the same year, the Centers for Disease Control and Prevention guidelines evaluated the mean cost caused by SSI treatment to be from $10,443 to $25,546 per SSI. This cost depends on many factors including the patient himself and the type of surgery.

**Conclusions:** Prevention of the risk of infection is, therefore, a profitable concept for surgery that must be integrated within all healthcare managements worldwide.

## Highlights

– Surgical site infections in digestive surgery generate an additional cost ranging from €306 to €26,815 per infected patient, depending on the depth and the comorbidities of the patient.
– The increased length of stay for digestive surgery-related infections ranges from 4 to 29 days.
– These data, therefore, encourage us to maintain a preventative approach both for the direct benefit of the patient and for the reduction of healthcare expenditure.

## Introduction

Surgical site infections (SSIs) represent the third most common source of nosocomial infections in France with an estimated prevalence of 0.83% (0.71–0.95) just after urinary tract and lower respiratory infections. SSIs have been on the rise, predominantly those affecting deep tissues, organs, and cavities (1).

Surveillance data shows an SSI rate of 1.97% for digestive surgery [95% CI = (1.81–2.13)]. The incidence varies depending on the type of intervention. Indeed, inguinal or crural hernia operations have an SSI rate of 0.6% [95% CI = (0.37–0.83)], while in colorectal surgery, the rate is 4.93% (2.91–6.94). In general, the level of SSI is higher than that found in other surgeries because the surgical field is heavily colonized by digestive flora (2).

These postoperative infections not only have consequences for the patient themselves but also have an economic impact on the healthcare system. It is, therefore, important to estimate the medico-economic impact of SSIs in general and in digestive surgery in particular.

Their consequences in terms of morbidity and mortality are often significant and their surgical or medical management (antibiotic therapy) is often complex.

Specialized and multidisciplinary structures have been setup to improve the treatment of these patients during complex SSIs, such as the reference centers for complex osteoarticular infections in 2008 in France called “CRIOA” (3, 4). This multidisciplinary approach is expanding into the management of other types of healthcare-associated infections due to changes in medical practice and the antibiotic resistance profile of bacteria.

The economic challenges for society are substantial and particularly important in the period of the economic crisis that we have been going through over the past several years. A recent report from the Office of the Auditor General considers that infections associated with healthcare represent globally “a cost (…) difficult to establish; a previous study places them in a range of 2.4 billion euros (1 to 6 billion)” (5).

This study aimed to produce a summary of the cost induced by the occurrence of these SSIs.

## Methodology

A literature review was carried out on PubMed and Syrtis using the following keywords: “hospital-acquired infection,” “hospital cost,” “economic impact,” “surgical site infection,” “length of stay,” “economic consequence,” “economic burden,” and “surgical wound” between 2009 and 2020.

All countries were included in this study regardless of their income status.

The following were excluded from this review of the literature:

- Publications in languages other than English or French;
- Articles relating to children due to pediatric specificity;
- Studies from the point of view of the patient; and
- Studies related to costs of society;

Costs described in the studies were borne by patients themselves or society/healthcare systems. Eligible articles included cost analysis with partial or complete economic evaluations. The costs have been converted into euros. They have been standardized in 2020 by consulting the history of currency rates on the site: https://fxtop.com/fr/historique-rate-change.php. For the given year, the currency rate taken is that on December 30, 2020.

When the studies did not relate specifically to digestive surgery, we individualized the specific costs of this surgery when the methodology of the studies identified allowed it.

## Results

About 21 studies met the inclusion criteria and were, therefore, analyzed to summarize the cost and the increase in the length of hospital stay associated with SSIs. The main results are shown in Table 1 below.

**Table 1:** Increase in duration and additional costs associated with surgical site infections (SSIs).

**Type of surgery**	**Additional average cost (euros) /episode [by SSI]**	**Increase of hospital stay (days)[sum of days for the rehospitalization]**	**Country**	**Reference**
**All**	4,490–24,514	2.9–54	FR, GER, ITA, ES, UK	Badia et al. (6)
**All**	2,857–46,570	4.9–32.2	NL	Broex et al. (7)
**All**	7,262	4.26	USA	Boltz et al. (8)
**Digestive**	7,330	–	UK	Guest et al. (9)
**Digestive**	12,703–14,736	8.9–10.0	USA	De Lissovoy et al. (10)
**Digestive**	3,540–26,815	4–29	UK	Jenks et al. (11)
**Digestive**	2,430–3,640	15	FRA	Lamarsalle et al. (12)
**Digestive**	306–2,281	2.8–9.5	JPN	Fukuda et al. (13)
**Digestive**	7,159–9,703	4.6–7.9	USA	Keenan et al. (14)
**Neurosurgery**	8,177–22,344	3–7	USA	Patel et al. (15)
**Neurosurgery**	14,262	6.9–9.6	USA	Blumberg et al. (16)
**Neurosurgery**	9,002	–	UK	Atkinson et al. (17)
**Cardiac**	19,661	–	UK	Chiwera et al. (18)
**Cardiac**	2,670–28,062	–	GER	Graf et al. (19)
**Cardiac**	24693	48–49.7	JPN	Kobayashi et al. (20)
**Orthopedic**	8,581–13,108	5.7–9.2	GER	Poultsides et al. (21)

*GER, Germany; ITA, Italy; ES, Spain; UK, United Kingdom; NL, Netherlands; US, United States; JPN, Japan*.

We note that the cost range related to SSIs in digestive surgery is €306–26,815 vs. €2,610–46,570 for other types of surgery. Similarly, the increase in the length of stay associated with SSIs in digestive surgery ranges from 2.8 to 29 days vs. 2.9–54 days for other types of surgery.

Regarding low- and middle-income countries, five studies have been found: China (*n* = 2) (22, 23), Jordan (*n* = 1) (24), Brazil (*n* = 1) (25), and India (*n* = 1) (26). Additional cost of SSIs ranged from $3865 (Brazil) to $29,610 (India).

## Discussion

This study highlighted that most of the costs of SSIs treatment (without taking into consideration the cost of initial surgery) are borne by surgical revision (37.9% of the total cost) and by the need for rehospitalization (59.4%) (15, 17). However, it is important to stress out that additional costs to manage SSIs arise from medical staff and investigations (27).

This analysis made it possible to identify a range of costs directly induced by the occurrence of an SSI. The variations observed are sometimes significant and this is explained by:

- Structural reasons linked to the country (and, therefore, to the healthcare system); but no differences were found regarding the status of the country by itself (high income vs. low/middle income) in accordance with the recent study of Monahan et al. (28). However, it seems important to underline that few studies have been found for low-/middle-income countries and that they are as heterogeneous as those in Europe. In France, since 2018, with the “Surveillance and prevention of the risk of infection in surgery and interventional medicine” (SPICMI) mission, the most frequent surgical procedures in France are followed and their SSIs are recorded. Considering that this surveillance has the ambition to be fully automatized within the next few years, the evaluation of costs may be more efficient and accurate in accordance with the European Centre for Disease Prevention and Control in the future.
- Methodological aspects: First, with the definition of SSI: most of the studies use the definition of the Center of Disease Control (CDC) for SSI but do not apply it; moreover, follow-up is not setup for some studies missing post-discharge SSIs. Second, with the analysis: the type of surgery (specific or mixed), patient population, and cost items including analysis with or without confounders are very heterogeneous.

Indeed, the comparison of health systems and their financing does not allow a homogeneous treatment of the data resulting from these studies. The point of view of the study is also a factor that can make the analysis complex: the cost will not be the same if we look from the point of view of society, from the point of view of the hospital, and from the point of view of the paying agency or the patient. In the articles reviewed for this study, we excluded studies whose point of view was that of the patient or society (not considering indirect costs).

In addition, some studies have included a wide variety of surgical procedures, which may reinforce the heterogeneity of the data in the pooled economic analysis. Thus, we observe that SSIs occurring after cardiac surgery or neurosurgery have an overall greater economic impact than SSIs occurring after cutaneous or gynecological surgery (6, 7, 15). The cost induced by SSIs in digestive surgery is in a rather high range compared to other surgeries.

Finally, the bacterial causative agents responsible for SSIs, regardless of surgery, do not all have the same pathogenicity and, therefore, the same economic consequences (8).

Patient comorbidities also influence the economic impact of an SSI (29, 30). However, some studies do not analyze their result taking into consideration these potential cofounders.

Similarly, the type of SSI is not always specified in the studies. When we had the information, we could see that the cost of an SSI is dependent on the depth of the infection. It has been observed that the deeper the infection, the greater was the economic consequences.

Thus, in digestive surgery, the cost varies by a factor of 1 (superficial SSI) to 2.3 (organ SSI), and the length of stay attributable to the SSI of the organ increases by a factor of 3.4 (13). There is a link between the increased length of stay associated with the infection and the observed additional cost. This almost linear relationship should be analyzed with the same limitations as those mentioned above: factors related to the patients, the depth of the infection, and the type of surgery (8, 10).

As a result, this study shows the challenges toward assessing with accuracy the costs of SSIs as huge range costs have been identified between the studies. We have, thus, the main limitation of the study, which can be explained by different factors such as differences in follow-up between the studies, non use of the definition of SSI according to the CDC (implying a minimum of 30 days of follow-up of surgery), the absence of adjustment for potential confounders in some studies, and heterogeneity within studies highlighting the lack of robustness of studies.

## Conclusion

Surgical site infections are responsible for a cost that is very likely >€1 billion (it is the second most common cause of nosocomial infection which costs at least €2–4 billion per year in France). This report has shown that this cost depends on many factors including the patient himself and the type of surgery: the more the type of surgery is contaminated, the more the risk of SSI increases, leading to digestive surgery as one of the type of surgeries at risk of SSIs.

Beyond the expected benefit for the patient, this report can and should serve as new arguments for public authorities to invest in the prevention of SSIs. An old and recent study on the cost-effectiveness analysis of preventive measures in surgery can be found in the literature (31–33).

Given the economic resources that are depleted, the prevention of the risk of infection is, therefore, a profitable concept for surgery that must be put forward.

## Author Contributions

EP, NC, and EL conceived and designed the study. EP acquired the data. EP, EL, JR-Y, PB, and NC analysed the data. EP, EL, JR-Y, PB, and NC interpreted the data. EP, EL, JR-Y, PB, and NC contributed to the writing of the manuscript and to its critical revision. All authors contributed to the article and approved the submitted version.

## Conflict of Interest

The authors declare that the research was conducted in the absence of any commercial or financial relationships that could be construed as a potential conflict of interest.

## Publisher's Note

All claims expressed in this article are solely those of the authors and do not necessarily represent those of their affiliated organizations, or those of the publisher, the editors and the reviewers. Any product that may be evaluated in this article, or claim that may be made by its manufacturer, is not guaranteed or endorsed by the publisher.
